# The Bacteriology of Inflammatory Processes in the Abdomen

**Published:** 1904-12-10

**Authors:** 


### THE BACTERIOLOGY OF INFLAMMATORY PROCESSES IN THE ABDOMEN.

Dr. J. T. Hewetson, a research scholar of the British Medical Association, has recently been engaged in an investigation, the principal object of which was to discover what particular micro-organisms, if any, could be found associated with inflammatory processes originating in various abdominal lesions. The report which embodies the results of his work up to the present date contains several interesting features.

### Appendicitis.

The cases of appendicitis examined are divided into those not accompanied by suppuration and those in which suppuration was present. In the non-suppurative forms, of which there were 44 cases, cultivations were attempted with material removed from the proximal end of the excised appendix, and also from the cavity of the appendix near the tip. In 25 of the 44 cases the appendix was completely stenosed. Of these cases the contents of the distal chamber were sterile in 11. In the 14 which were not sterile, bacillus coli alone was found in 11. Of the 19 cases showing no stenosis 18 gave a growth (bacillus coli alone in 16) and 1 was sterile. In the 44 appendices 4 concretions were found.

From facts observed in cases of suppurative appendicitis Dr. Hewetson concludes that there is strong evidence in favour of the view that organisms not infrequently die in an abscess cavity previous to its evacuation. In the suppurative cases, as in the others, the bacillus coli was by far the most common organism found. Actinomycosis was found in one case, and was present not only in the pus around the appendix, but in the liver also.

### Cholecystitis.

In only 3 out of 19 cases of gall-stones were the contents of the gall-bladder sterile, Dr. Hewetson's observations being thus in accord with those of previous workers in the same field. Further tests showed that a number of different organisms could be grown as profusely in sterile human bile as in peptone broth, and that human bile possesses little or no bactericidal property.

### Gastric Ulcer.

Some unexpected results were arrived at by bacteriological examinations of the contents of the stomach and duodenum obtained during operations on these organs. As the patients had not taken any food for some hours before the operation, the observations made only apply, as Dr. Hewetson is careful to point out, to the fasting stomach. He found that in gastric dilatation without ulceration the stomach was sterile in 83 per cent., while in gastric dilatation with ulceration, the stomach contents were sterile in only 28 per cent.—a marked difference and one which may not be without some practical significance. Further, Dr. Hewetson tested the power of the stomach to destroy micro-organisms introduced into its cavity. These experiments were performed upon himself and upon patients who had undergone gastrostomy. By swallowing virulent cultures from cases of acute osteomyelitis and pyæmia and afterwards removing small quantities of the gastric contents by means of a stomach tube passed every 15 minutes, he found that the ordinary pyogenic cocci were killed in from 30 to 45 minutes, and bacilli were killed in an hour or an hour and a half. The relative sterility of the stomach which these experiments show is quite in accordance with surgical experience. As Dr. Hewetson remarks, if general peritonitis follows operation upon the stomach and jejunum, it will almost always be due to infection from without and not from the viscera themselves. This position is strengthened by the fact that cultivations made of the fluid in the general peritoneal cavity in cases of perforation of the stomach or duodenum showed these contents to be sterile in four out of ten cases, although in two out of the four the perforation had taken place 24 hours previously.

We shall look forward to Dr. Hewetson's further communications, with pleasant anticipation, for the field of his present labours is one which should have been thoroughly explored years ago—the more so as it is one of great surgical importance.

